# Strategies and Opportunities to Improve Community Health through Advanced Molecular Detection and Genomic Surveillance of Infectious Diseases

**DOI:** 10.3201/eid3113.241408

**Published:** 2025-05

**Authors:** Jazmyn Moore, Ruth Sanon, Yury Khudyakov, Nathelia Barnes

**Affiliations:** Author affiliation: Centers for Disease Control and Prevention, Atlanta, Georgia, USA

**Keywords:** community health, epidemiology, surveillance, public health, disease outbreaks, infectious disease outbreaks, epidemiologic methods, public health practice, bioinformatics, informatics, public health informatics, genomics, whole-genome sequencing, COVID-19, respiratory infections, severe acute respiratory syndrome coronavirus 2, SARS-CoV-2, SARS, coronavirus disease, zoonoses, viruses, coronavirus, HIV/AIDS and other retroviruses, sexually transmitted infections, tuberculosis and other mycobacteria, bacteria

## Abstract

Advanced molecular detection (AMD) refers to the integration of next-generation sequencing, epidemiologic, and bioinformatics data to drive public health actions. As new AMD technologies emerge, it is critical to ensure those methods are used in communities that are most affected by disease-induced illness and death. We describe strategies and opportunities for using AMD approaches to improve health in those communities, which include improving access to pathogen sequencing, increasing data linkages, and using pathogen sequencing for those diseases where sequencing technologies can provide the best health outcome. Such strategies can help address and prevent differences in health outcomes in various populations, such as rural and tribal communities, persons with underlying health issues, and other populations that experience higher risks for infectious disease.

Advanced molecular detection (AMD) is the integration of next-generation pathogen sequencing, epidemiologic, and bioinformatics data to enable disease identification, responses, and public health actions (*1*). Specimens collected through public health activities, such as routine surveillance and outbreak investigations, are used by laboratory scientists to perform genomic sequencing of pathogens. Bioinformaticians and data scientists analyze and link the large amounts of genetic information obtained from pathogen DNA sequencing with epidemiologic and other data to better elucidate the origin and characteristics of a pathogen, including its potential response to countermeasures such as antiviral medications. This information is then shared with epidemiologists who use the data to determine transmission patterns and to identify and stop outbreaks (*2*). Those methods have been used to detect and monitor pathogens that cause infectious diseases, including those pathogens disproportionately affecting certain populations, such as hepatitis C virus (HCV) (*3*), HIV (*4*), *Mycobacterium tuberculosis* (*5*), and more recently, SARS-CoV-2 (L. Lyu et al., unpub. data, https://doi.org/10.1101/2023.12.28.23300535).

Epidemiology, which drives public health efforts, is the scientific study involved in identifying which populations are affected by different health conditions and why (*6*). The COVID-19 pandemic highlighted differences in health outcomes across various populations and the need to address factors that caused those differences. Here, we discuss strategies and opportunities for improving community health through AMD and genomic surveillance of pathogens.

## Strategy 1—Ensuring Access to AMD Technologies

The US public health system has consistently faced funding challenges. For example, among local health departments, those located in rural areas are often understaffed and underfunded compared with their urban and suburban counterparts, hindering their ability to perform the 10 Essential Public Health Services originally enumerated in 1994 and updated in 2020 (https://www.cdc.gov/public-health-gateway/php/about/index.html) (*7*). Because of a series of factors, including challenges with accessing care, socioeconomic status, and living and working conditions, persons living in rural areas can have more barriers to care and worse health outcomes.

The Centers for Disease Control and Prevention (CDC) provides funding to health departments in all US states, territories, and freely associated states and in 7 large cities to increase each jurisdiction’s capacity to build and integrate laboratory, bioinformatics, and genomic epidemiology technologies as part of their infectious disease prevention capabilities (*8*). Technical assistance for AMD technologies can be provided to areas with fewer resources to modernize disease investigation techniques and can promote rapid public health responses; AMD methods provide faster, cost-effective results compared with older sequencing methods (*2*).

Although pathogen genome sequencing and other AMD technologies can expedite critical public health advances, it is critical to ensure that medically underresourced communities, including rural areas, are not left behind when new health technologies are adopted. When areas and communities with more resources are able to access health interventions that are generally inaccessible to groups with less resources, then existing differences in health outcomes can increase.

### Example—Providing Support to Understand SARS-CoV-2 Transmission in Rural Texas

One method to distribute the benefits of AMD technologies is through larger health departments providing sequencing support to smaller health departments and communities that have no on-site sequencing or bioinformatics capacity; this support would aid outbreak investigation and surveillance efforts. For example, the Houston Health Department in Houston, Texas, USA, and academic partners used CDC funds and technologies to perform phylogeographic inference (a method that enables scientists to map how pathogens spread through space and time) to determine SARS-CoV-2 transmission in a rural community. The investigators were able to determine that SARS-CoV-2 outbreaks in rural areas were driven by repeated introductions of the virus from urban centers (L. Lyu et al., unpub. data). Those findings can help guide public health actions, such as resource allocation, contact tracing, and prevention efforts.

### Opportunity—Improving Data Linkages

Factors influencing infectious disease outcomes can be better determined by ensuring relevant accompanying data, such as social, environmental, and demographic variables, are collected and linked to samples. Those data can be analyzed alongside test results and can highlight patterns of infectious disease transmission and identify populations at increased risk for illness and death. Integrating those data elements into systems and analyses can be done by strengthening linkages between epidemiologists and laboratory scientists. CDC has previously emphasized the importance of data integration and has supported efforts to increase staffing of personnel who perform those functions, such as laboratory scientists, genomic epidemiologists, data scientists, and bioinformaticians.

## Strategy 2—Considering Public Health Effects When Prioritizing Pathogens for AMD Technologies

When public health resources and capacity are limited, outbreaks and public health problems are often assigned different levels of priority for funding and human resources. A strategy to reduce differences in health outcomes is to use pathogen genomic sequencing for populations disproportionately affected by illness or death when prioritizing those pathogens. Examples of pathogens that disproportionately affect certain populations are HIV, HCV, and *M*. *tuberculosis*.

### Example—HIV in Men Who Have Sex with Men

In the United States, HIV has historically and disproportionately affected men who have sex with men (MSM), a population that also experiences social stigma and discrimination. AMD has been used to elucidate HIV transmission patterns to inform prevention, response, and public health program efforts (*4*). For example, the Georgia Department of Public Health, Atlanta, Georgia, USA, a part of CDC’s Pathogen Genomics Center of Excellence, has used molecular cluster detection in routine surveillance to better determine HIV transmission patterns. The investigation found that Hispanic/Latino MSM were disproportionately represented in new HIV infections (*4*). That information guided subsequent qualitative investigations that revealed gaps in HIV prevention service coverage and informed culturally responsive HIV prevention activities focused on Hispanic/Latino MSM.

Some networks of persons living with HIV have raised concerns about using genomic surveillance for HIV prevention (*9*). Concerns have focused on privacy, confidentiality, consent, stigma and institutional bias, and criminalization (*9*). When conducting genomic surveillance, particularly for infectious diseases such as HIV and mpox that have been stigmatized (*10*,*11*), CDC prioritizes ethical and credible practices and advises all funded health departments to also uphold those practices. When considering implementation, the benefits of genomic surveillance activities should outweigh the risks; risks and benefits should be clearly communicated to potentially affected populations through community and clinical partners at the point of care and during other opportunities to engage affected persons. In addition to community engagement, other ethical considerations, including data use agreements, plans for data storage, and data destruction protocols, should be evaluated and incorporated into surveillance plans (*12*,*13*). CDC has supported a series of information technology features that address data security and limit the misuse of any related AMD data (*1*). Finally, it is critical to communicate the actions taken to safeguard data and avoid unintentionally discouraging persons from seeking care (*14*). When possible, a collaborative approach should be taken that involves community members and community-based organizations to ensure that surveillance and outbreak investigation activities are conducted in a way that does not cause harm.

### Example—Understanding HCV Transmission in Rural Indiana

Hepatitis C is another infectious disease that disproportionately affects certain populations and for which AMD has been used to guide public health action. Since 2004, cases of acute HCV infection associated with injected drug use have increased; persons who inject drugs are at highest risk for infection (*3*). An investigation of HIV and HCV infections among persons in rural Indiana who injected drugs revealed that widespread circulation of numerous HCV strains occurred long before an HIV outbreak began (*15*). A cloud-based AMD toolkit, the Global Hepatitis Outbreak and Surveillance Technology, helped detect a large HCV transmission network, which enabled the HIV outbreak (*15*). The HCV transmission network data were used to inform the development of tailored public health interventions to efficiently interrupt HCV transmission among persons who inject drugs (*16*). Results from the investigation suggested HCV transmission could be used as a warning sign for eventual HIV transmission, and HIV surveillance among persons with HCV infection and those who inject drugs could help to rapidly identify and halt both HIV and HCV transmission. The interruption of HIV and HCV transmission has critical health and economic implications, particularly in rural settings where access to healthcare might be limited. The results also underscored the need to increase knowledge of and access to HCV testing and treatment services among persons who inject drugs to prevent further disease transmission.

### Example—Tuberculosis in Non–US-Born Populations

Tuberculosis (TB) disproportionately affects persons with HIV, those who report substance use, non–US-born persons within the United States, and persons with certain medical conditions, such as diabetes (*17*). Whole-genome sequencing is crucial to determine TB transmission networks and to detect drug-resistant cases, which require special treatment protocols (*18*). In addition, CDC developed MicrobeTrace, a web-based AMD tool, that helps users visualize genomic relationships and epidemiologic links to help track outbreaks of tuberculosis and other diseases (*19*). In 2022, most (96%) isolates from culture-positive TB cases were sequenced; this information was routinely analyzed, and the output was made available to public health partners for programmatic follow-up, including allocation of resources for investigation and intervention for specific cases (*17*). AMD technologies play a critical role in the identification and treatment of TB in the United States and globally.

In addition to considering traditional pathogen attributes, such as transmissibility and disease severity, prioritizing pathogens that disproportionately affect certain populations can help reduce health disparities. Priorities should be determined by jurisdictions and monitored and regularly reevaluated to ensure that they adequately address the most pressing public health challenges.

Across various populations and pathogens, it is critical to address privacy concerns, appropriately inform affected communities of applicable findings, and to use findings to optimize prevention efforts. To address those considerations, the AMD program relies on strong relationships between laboratory scientists and epidemiologists in health departments. Those relationships enable strategic interventions, engagement with communities, and strong disease outbreak investigations that promote more efficient and effective public health action.

## Strategy 3—Ensuring a Robust Public Health Workforce

Public health workforces having varied training, expertise, and backgrounds can better address the needs of the communities they serve (*20*). Workforce heterogeneity across various dimensions permits the inclusion of differing opinions and perspectives and can improve organizational success (*21*).

### Example—CDC Fellowships

Through various training opportunities, CDC supports the development of highly qualified and talented persons across the public health workforce, including those supporting AMD. The CDC’s Office of Advanced Molecular Detection, National Center for Emerging and Zoonotic Infectious Diseases, has promoted the use of fellowships and collaborations with nontraditional partners, such as universities and other academia, to increase awareness of AMD practices and expand the skill and expertise of the AMD workforce in both laboratory and health department settings. The Office of Advanced Molecular Detection has also demonstrated a long-standing commitment to training a professionally diverse cohort of next generation public health laboratory scientists through bioinformatics and genomic epidemiology fellowships in partnership with the Association of Public Health Laboratories. Open to students with varied academic and sociodemographic backgrounds, those programs are critical for providing highly qualified talent to the public health workforce.

### Opportunity—Training

Training on how to collaborate with colleagues of various backgrounds, including topics such as working across generations and backgrounds and effective communication, can help generate cohesiveness in the workforce. For epidemiologists, training topics of interest might include collecting and analyzing demographic data variables, precisely measuring health outcomes in emerging genomic applications across different communities, and working with communities. For laboratory scientists, training topics of interest might include sampling strategies, data quality and timeliness, and using electronic health records as tools to identify and reduce differences in health outcomes. For bioinformaticians, training topics of interest might include recognizing and minimizing bias in algorithm design, privacy, and ethics.

It is critical that members of the workforce understand their roles in improving public health and the importance of integrating AMD technologies and approaches to reduce disease occurrence. As we better train our workforce, opportunities exist to create more effective public health interventions, multipathogen assessments, and guidelines to monitor and manage complex pathogens that affect communities.

## Moving Forward

Without special consideration when introducing and expanding innovative public health technologies, existing disparities in infectious disease risk can be unintentionally exacerbated, and new disparities can arise. Public health efforts should consider populations that are disproportionately affected by illness or death caused by infectious diseases and mitigate disparate health outcomes in those groups. This mitigation can be achieved by prioritizing AMD resources, such as for rural or medically underserved areas or for pathogens that disproportionately affect different communities. Ensuring that the public health workforce reflects the population that it serves can improve overall effectiveness of control and prevention efforts. An ever-evolving field, AMD is transforming public health activities, deepening our knowledge of disease transmission and pathogen resistance mechanisms, and helping to resolve outbreaks. Ensuring that AMD technologies are used to close gaps in disparate health outcomes and prevent new gaps from arising will require strategic planning and implementation, community engagement, continued monitoring, and sustained resources.
